# Diazoxide Maintains Human Myocyte Volume Homeostasis During Stress

**DOI:** 10.1161/JAHA.112.000778

**Published:** 2012-04-24

**Authors:** Sara K. Maffit, Angela D. Sellitto, Ashraf S. Al-Dadah, Richard B. Schuessler, Ralph J. Damiano, Jennifer S. Lawton

**Affiliations:** Division of Cardiothoracic Surgery, Department of Surgery, Washington University School of Medicine, St. Louis, MO

**Keywords:** myocardial stunning, stress, surgery

## Abstract

**Background:**

Exposure to hypothermic hyperkalemic cardioplegia, hyposmotic stress, or metabolic inhibition results in significant animal myocyte swelling (6% to10%) and subsequent reduced contractility (10% to 20%). Both are eliminated by the adenosine triphosphate-sensitive potassium channel opener diazoxide (DZX). The relationship between swelling and reduced contractility suggests that the structural change may represent one mechanism of postoperative myocardial stunning. This study evaluated human myocyte volume during stress to investigate if similar phenomena exist in human myocytes.

**Methods and Results:**

Human atrial myocytes isolated from tissue obtained during cardiac surgery were perfused with Tyrode's physiological solution (20 minutes, 37°C), test solution (20 minutes), and Tyrode's (37°C, 20 minutes). Test solutions (*n*=6 to 12 myocytes each) included Tyrode's (37°C or 9°C), Tyrode's+DZX (9°C), hyperkalemic cardioplegia (9°C)±DZX, cardioplegia+DZX+HMR 1098 (sarcolemmal adenosine triphosphate-sensitive potassium channel inhibitor, 9°C), cardioplegia+DZX+5-hydroxydeconoate (mitochondrial adenosine triphosphate-sensitive potassium channel inhibitor, 9°C), mild hyposmotic solution±DZX, metabolic inhibition±DZX, and metabolic inhibition+DZX+5-hydroxydeconoate. Myocyte volume was recorded every 5 minutes. Exposure to hypothermic hyperkalemic cardioplegia, hyposmotic stress, or metabolic inhibition resulted in significant human myocyte swelling (8%, 7%, and 6%, respectively; all *P*<0.05 vs control). In all groups, the swelling was eliminated or lessened by DZX. The addition of channel inhibitors did not significantly alter results.

**Conclusions:**

DZX maintains human myocyte volume homeostasis during stress via an unknown mechanism. DZX may prove to be clinically useful following the elucidation of its specific mechanism of action. **(*J Am Heart Assoc*. 2012;1:jah3-e000778 doi: 10.1161/JAHA.112.000778.)**

## Introduction

Previously, our laboratory documented the detrimental consequences of three stresses (metabolic inhibition [MI], mild hyposmotic stress, and exposure to hypothermic hyperkalemic cardioplegia) on myocyte volume and contractility in isolated animal (rabbit and mouse) myocytes.^1–4^ All three stresses resulted in significant myocyte swelling (6% to 10%) and an associated decline in contractility (10% to 20%) in both species. In both species, diazoxide (DZX, adenosine triphosphate-sensitive potassium (K_ATP_) channel opener) prevented the detrimental swelling.^1–4^

Interestingly, the prevention of the detrimental myocyte swelling (structural derangement) resulted in elimination of the associated reduced contractility (functional derangement). We have proposed that volume derangement and function are inversely related and that myocyte swelling secondary to stress may represent one mechanism of myocardial stunning.^4^

Openers of the K_ATP_ channel have been demonstrated to limit ischemic injury, preserve myocardial function, mimic ischemic preconditioning, and alleviate the effect of myocardial stunning in multiple models.^5–9^ However, the mechanism of cardioprotection is unknown. Multiple theories exist regarding the proposed mechanism of cardioprotection provided by K_ATP_ channel openers. Theories implicate both the sarcolemmal and purported mitochondrial K_ATP_ (mK_ATP_) channels as well as K_ATP_ channel-independent effects of the pharmacological openers themselves.^10–14^ The cardioprotection observed by opening the K_ATP_ channel, or the channel openers themselves, may involve the maintenance of volume homeostasis (and contractility) at the myocyte level.

The current study was devised to establish the relevance of observations in animal tissue to those in human tissue to support subsequent use in humans. This study was performed to determine if human myocytes undergo similar significant swelling secondary to exposure to hyperkalemic cardioplegia, mild hyposmotic stress, and MI and if the K_ATP_ channel opener, DZX, would prevent this detrimental consequence.

## Methods

Experiments were approved by the Washington University Human Studies Committee in accordance with Institutional Review Board approval and patients gave informed consent for participation.

### Human Myocyte Isolation

Normally discarded tissue specimens (right or left atrium) were collected during elective cardiac surgery with patient consent. Tissue was immediately placed into 37°C oxygenated calcium-free Tyrode's physiological solution (Tyr) (in mmol/L): NaCl 130, KCl 5, KH_2_PO_4_ 0.4, MgCl_2_ 3, HEPES (N-[2-hydroxethyl]piperazine-N′-[4-butanesulfnonic acid]) 5, taurine 15, glucose 10, and creatine 5.7 (pH adjusted to 7.3 by 20% NaOH titration), Na_2_EGTA 0.1, and nitrilotriacetic acid 6 (NTA, Sigma Chemical Corporation, St. Louis, MO) and taken immediately to the laboratory (5 minutes), where they were placed into fresh oxygenated 37°C calcium-free Tyr with 0.1 mmol/L Na_2_EGTA and 6 mmol/L NTA, and minced. Minced tissue was transferred to a flask containing 20 mL of 37°C calcium-free Tyr with 6 mmol/L NTA and agitated in a 37°C water bath at 100 rpms for 12 minutes to remove extracellular calcium. Tissue was then transferred to a flask containing 10 mL of 37°C calcium-free Tyr with 1000 mg/L bovine serum albumin (Sigma Chemical Corporation), 925 mg/L collagenase type II (Worthington Biomedical, Freehold, NJ), and 250 mg/L protease (Sigma Chemical Corporation) and agitated in a 37°C water bath at 100 rpms for 70 minutes. This solution was then centrifuged at 100*g* at 4°C for 5 minutes. The supernatant was discarded and the pellet resuspended in 15 mL of 37°C calcium-free Tyr with 1000 mg/L bovine serum albumin (Sigma Chemical Corporation) and 925 mg/L collagenase type II (Worthington Biomedical) and agitated in a 37°C water bath at 100 rpms for 20 minutes. This solution was then centrifuged at 100*g* at 4°C for 5 minutes. The previous two steps were then repeated. The supernatant was discarded and the pellet resuspended in a 37°C cell isolation solution containing (in mmol/L): potassium glutamate 120, KCl 10, KH_2_PO_4_ 10, MgSO_4_ 1.8, K_2_EGTA 0.5, taurine 10, HEPES 10, and glucose 20, and triturated to separate the cells. This solution was then filtered through 300 micron nylon mesh to remove large debris and centrifuged at 100*g* at 4°C for 10 minutes. The supernatant was discarded and the pellet resuspended in cell isolation solution and centrifuged again at 100*g* at 4°C for 10 minutes, three times. The supernatant was then discarded and the pellet resuspended in cell isolation solution and allowed to settle for 30 minutes.^15–17^

### Myocyte Imaging

Myocytes were used immediately on the day of isolation and were not cultured. Myocytes were visualized on a slide on a glass-bottom chamber on an inverted microscope stage (Leitz, Wetzlar, Germany) as previously described.^4^ An aliquot of the isolated cells was placed into the chamber and allowed to stabilize for 5 minutes, after which the chamber was perfused at a rate of 3 mL/min with Tyrode's physiological control solution (in mmol/L): NaCl 130, KCl 5, CaCl_2_ 2.5, MgSO_4_ 1.2, NaHCO_3_ 24, Na_2_HPO_4_ 1.75, and glucose 10 (buffered to a pH of 7.4 using 95% O_2_ to 5% CO_2_). Cells were evaluated for viability based on the following criteria: normal rod shape, smooth edges, sharp borders, clear striations, absence of blebbing, and lack of spontaneous contractions.^18^ Only viable cells were used. Cell length, width, and area were manually traced using Scion Image software (Scion Corporation, Frederick, MD) and estimated as previously described.^4,18^

### Experimental Protocol

Cells were perfused for 20 minutes with 37°C control Tyr to obtain baseline volume. Any changes in cell volume secondary to the isolation or imaging protocol would be evident during this period. Myocytes were then perfused for 20 minutes with test solution followed by a 20 minutes reexposure period with 37°C control Tyr. Test solutions included control Tyr (Tyr 37°C, *N*=12), Tyr 9°C (*N*=12), Tyr+100 μmol/L DZX (Sigma Chemical Corporation) (Tyr+DZX 9°C, *N*=12), St. Thomas hyperkalemic cardioplegia (Plegisol; Abbott Laboratories, North Chicago, IL) (St. T 9°C, *N*=12), St. T+100 μmol/L DZX (St. T+DZX 9°C, *N*=12), St. T+100 μmol/L DZX+40 μmol/L HMR 1098 (Aventis Pharma Deutschland Gmbh, Frankfurt, Germany) (St. T+DZX+HMR 1098 9°C, *N*=12), St. T+100 μmol/L DZX+50 μmol/L 5-hydroxydeconoate (5-HD, Sigma Chemical Corporation) (St. T+DZX+5-HD 9°C, *N*=12), altered Tyr (1T 37°C, control for osmotic experiments, *N*=9), hyposmotic solution (0.9T 37°C, *N*=9), hyposmotic solution+100 μmol/L DZX (0.9T+DZX 37°C, *N*=9), MI (37°C, *N*=6), MI+100 μmol/L DZX (MI+DZX 37°C, *N*=6), and MI+100 μmol/L DZX+50 μmol/L 5-HD (MI+DZX+5-HD 37°C, *N*=6).

St. Thomas hyperkalemic cardioplegia consisted of (in mmol/L): NaCl 110, NaHCO_3_ 10, KCl 16, MgCl_2_ 16, and CaCl_2_ 1.2, and was equilibrated with 95% O_2_ to 5% CO_2_ and titrated to the pH of 7.3 with 10% NaHCO_3_ solution. The addition of DZX did not significantly alter solution osmolarity (298±5 mOsm).^3^

Altered Tyrode's solution (1T) served as the control for the hyposmotic stress experiments. The 1T solution was made by substituting 130 mmol/L D-mannitol for 130 mmol/L NaCl in normal Tyrode's solution. The hyposmotic solution (0.9T) was made by substituting 97.5 mmol/L D-mannitol for 130 mmol/L NaCl in normal Tyrode's solution. This solution was used as this degree of hyposmotic stress provided a similar amount of myocyte swelling as that of hyperkalemic cardioplegia in animal studies, and this degree of swelling was ameliorated by DZX in animal myocytes.^2^

The MI solution provided complete MI, and this solution has been documented to provide significant myocyte swelling in animal myocytes.^4^ The solution consisted of (in mmol/L): NaCl 130, KCl 5, CaCl_2_ 2.5, MgSO_4_ 1.2, NaHCO_3_ 24, 2 deoxyglucose 10, and NaCN 2.

DZX is a purported mK_ATP_ channel opener. The dose of 100 μmol/L ameliorated cell swelling secondary to stress in previous animal studies.^1–4^ HMR 1098 is a purported specific sarcolemmal K_ATP_ (sK_ATP_) channel inhibitor and it was added to hyperkalemic cardioplegia in an attempt to eliminate any sK_ATP_ channel activity. 5-HD is a purported specific mK_ATP_ channel inhibitor and it was added during exposure to hyperkalemic cardioplegia and to MI in an attempt to eliminate any mK_ATP_ channel activity. The dose of 100 μmol/L DZX, 50 μmol/L 5-HD, and 40 μmol/L HMR 1098 were chosen based on previous experiments using animal myocytes.^1,4^ Stock solutions were made by dissolving 5-HD in double deionized water, HMR 1098 in double deionized water, and DZX in 0.1% dimethyl sulfoxide. Dimethyl sulfoxide has no effect on cell volume.^18^

The perfusion temperature of 37°C for control Tyr was chosen to mimic normal physiological temperature. The perfusion temperature of 9°C was chosen as it approximates myocardial temperature during cardioplegic arrest and is the temperature at which animal myocytes exhibited significant swelling during exposure to hyperkalemic cardioplegia. Myocytes were not subjected to any period of ischemia or modified ischemia as to eliminate any confounding cellular changes caused by ischemic stress.

Volume estimations were made after 5 minutes of exposure to control Tyr and every 5 minutes thereafter.

### Statistical Analysis

All data are presented as mean±standard error of the mean. A repeated measures analysis of variance was used for sequential time-based measurements for each test solution against its own baseline and control values. Using Fisher's least significant different test, post hoc multiple comparisons between different test groups were made separately during the baseline, test solution, and reexposure periods. Probability values <0.05 were considered significant. A Shapiro-Wilks test was used to test for normality. If the data failed the normality test, a nonparametric (Friedman's nonparametric repeated measures comparison) was used. Statistical analysis was preformed using SyStat 10.2 (SyStat Corporation, Point Richmond, CA).

## Results

**Figure 1.**
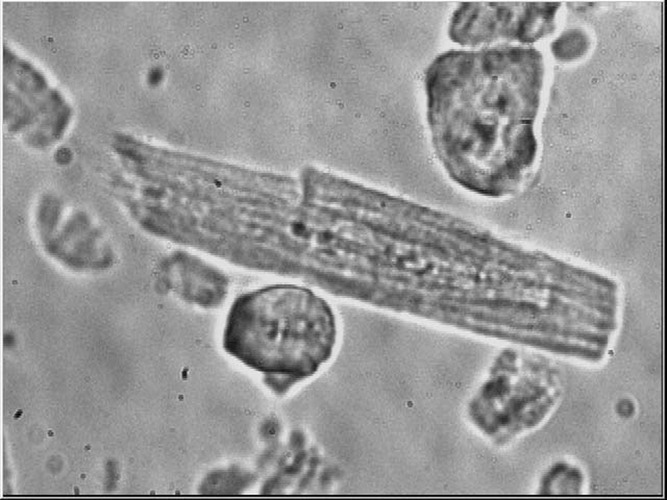
Representative isolated human myocyte surrounded by cellular debris at 40× magnification via inverted microscope.

Up to three myocytes per patient sample were used with cells randomly assigned to treatment groups. The yield of viable myocytes for human tissue was approximately 30% per field. A representative viable myocyte is depicted in Figure 1.

### Myocyte Volume

The mean length, width, and area of myocytes at baseline were 102.33±1.38 μm, 24.44±0.35 μm, and 2228.06±44.31 μm^2^, respectively. Assuming that the cross-section of the myocytes was a square, the mean volume of a single myocyte at baseline was 27.04±1.17 pico liter. However, if cells are actually cylindrical, this initial assumption would over estimate cell volume by a factor of 4/π or 1.27.^19^ To avoid this uncertainty, cell volume changes are presented relative to baseline values (which were calculated assuming the baseline volume was a square) taken during perfusion with control solution.

**Figure 2.**
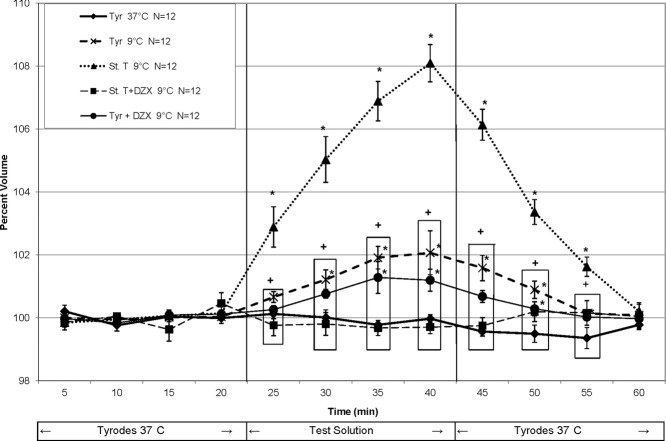
Diazoxide, a mitochondrial adenosine triphosphate-sensitive potassium channel opener, eliminates human myocyte swelling secondary to hypothermic hyperkalemic cardioplegia. After a 20 minutes period of exposure to control 37°C Tyrode's physiological solution (Tyr 37°C), isolated myocytes were exposed to test solution for 20 minutes (*n*=12 in each), and then reexposure to Tyr 37°C for 20 minutes. Cell volume change is represented as percent change from baseline (*y* axis) vs time (*x* axis). (*P<0.05 vs Tyr 37°C, +*P*<0.05 vs St. T 9°C).

During perfusion of control Tyr (37°C), cell volume remained stable throughout the entire experimental period (Figure 2). Perfusion with 9°C Tyr resulted in a significant increase in cell volume (2.06±0.69%, *P*<0.001 vs Tyr 37°C). On reexposure to Tyrode's at 37°C, cell volume returned to baseline (Figure 2). Perfusion with 9°C Tyr with DZX resulted in a lesser degree of cell swelling (1.19±0.35%, *P*<0.05 vs Tyr 37°C, *P*=NS vs Tyr 9°C). On reexposure to Tyrode's at 37°C, cell volume returned to baseline (Figure 2).

**Figure 3.**
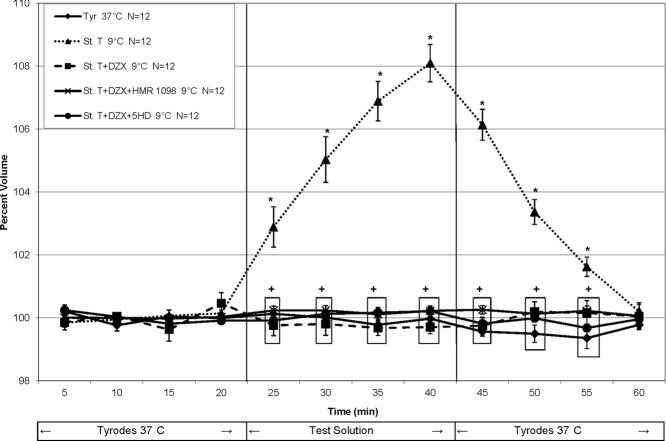
Sarcolemmal and mitochondrial adenosine triphosphate-sensitive potassium channel inhibitors do not alter human myocyte volume response to hyperkalemic cardioplegia in the presence of diazoxide. After a 20 minutes period of exposure to control 37°C Tyrode's physiological solution (Tyr 37°C), isolated myocytes were exposed to test solution for 20 minutes (*n*=12 in each), and then reexposure to Tyr 37°C for 20 minutes. Cell volume is represented as percent change from baseline (*y* axis) vs time (*x* axis). (*P<0.05 vs Tyr 37°C, +*P*<0.05 vs St. T 9°C).

Perfusion with 9°C St. Thomas hyperkalemic cardioplegic solution resulted in the greatest amount of cell swelling (8.09±0.59%; *P*<0.001 vs all other groups). On reexposure to Tyrode's at 37°C, cell volume returned to baseline (Figure 2). The addition of 100 μmol/L DZX to St. T 9°C eliminated the significant cell swelling (*P*=NS vs Tyr 37°C). The addition of 40 μmol/L HMR 1098 or 50 μmol/L 5-HD to St. Thomas+DZX 9°C did not alter cell volume (Figure 3).

**Figure 4.**
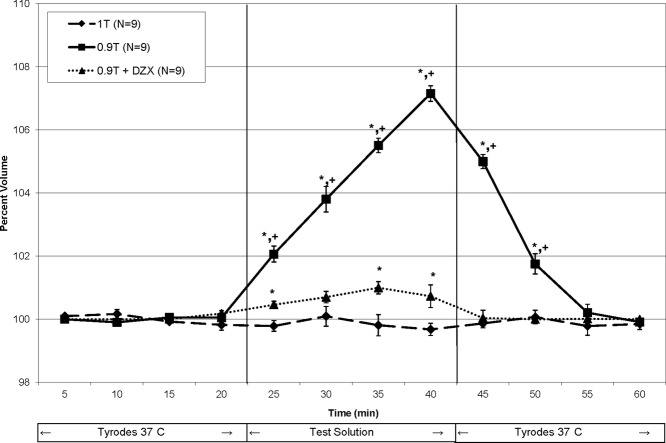
Mild hyposmotic stress results in significant human myocyte swelling that is ameliorated by the addition of diazoxide. After a 20 minutes period of exposure to control 37°C 1T Tyrode's physiological solution (1T 37°C), isolated myocytes were exposed to test solution for 20 minutes (*n*=9 in each), and then reexposure to 1T 37°C for 20 minutes. Cell volume is represented as percent change from baseline (*y* axis) vs time (*x* axis). (*P<0.05 vs 1T, +*P*< 0.05 vs 0.9T+DZX). 1T is altered Tyrode's solution (control) and 0.9T is hyposmotic solution (0.9 times normal).

During perfusion of 1T Tyr (control solution for osmotic alteration experiments), myocyte volume remained stable throughout the entire experimental period (Figure 4). During exposure to hyposmotic solution (0.9T), myocyte volume significantly increased (7.15±0.25%, *P*<0.05 vs 1T solution), and this was reduced to only a 1% volume increase by the addition of DZX (Figure 4).

**Figure 5.**
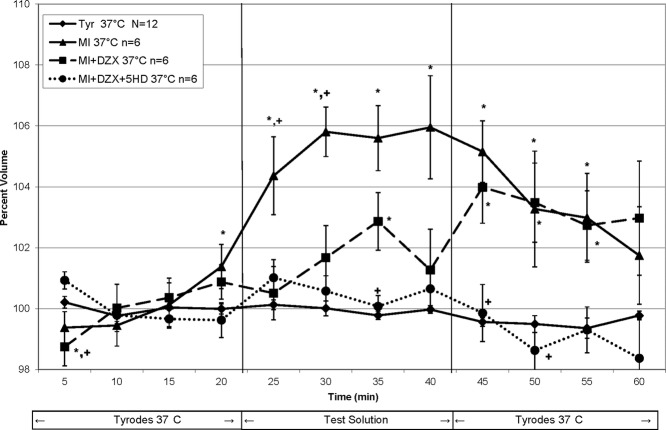
Metabolic inhibition results in significant human myocyte swelling that is ameliorated by the addition of diazoxide. After a 20 minutes period of exposure to control 37°C Tyrode's physiological solution (Tyr 37°C), isolated myocytes were exposed to test solution for 20 minutes (*n*=6 in each), and then reexposure to Tyr 37°C for 20 minutes. Cell volume is represented as percent change from baseline (*y* axis) vs time (*x* axis). (*P<0.05 vs Tyr, +*P*<0.05 vs MI+DZX).

During exposure to MI, myocyte volume significantly increased (5.95±4.15%, *P*<0.05 vs control), and this was reduced to only 1% to 3% volume increase with the addition of DZX (Figure 5). Cell volume in the MI+DZX group did not return to normal on reexposure to Tyr. Cell volume was not significantly different from control Tyrode's when 50 μmol/L 5-HD was added to the MI+DZX 37°C group (Figure 5).

## Discussion

Isolated animal myocytes demonstrate significant swelling and reduced contractility during exposure to hypothermic hyperkalemic cardioplegia, MI, or hyposmotic stress.^1–4,15,18^ DZX prevents these detrimental consequences secondary to all three stresses in two animal species.^1–4^ These detrimental consequences may be potentiated when the stresses are combined in situations such as cardiac surgery, and these changes may underlie one mechanism of postoperative myocardial stunning. This study was conducted to investigate if the same phenomena are observed in human myocytes.

This study confirmed that significant myocyte swelling occurs in isolated human myocytes secondary to exposure to hyperkalemic cardioplegia, hyposmotic stress, and MI. This significant swelling was eliminated or lessened by the addition of DZX (a known K_ATP_ channel opener) with or without pharmacological inhibition of the K_ATP_ channel. This confirmation of responses in human myocytes is vital to any future translation to clinical use.

Hypothermic hyperkalemic cardioplegia or exposure to hyposmotic stress results in myocyte swelling because of exposure to a hyposmolar extracellular environment. In contrast, MI results in myocyte swelling because of the development of a hyperosmolar intracellular environment. Interestingly, DZX (by an unknown mechanism) provides cellular volume homeostasis by lessening or eliminating myocyte swelling during exposure to all three stresses.

It is not known if the beneficial effect of DZX observed in isolated myocytes is related to cardioprotective effects that have been documented at the whole organ or the organism level. We propose that myocyte swelling (which we have shown to be associated with decreased contractility) may be one mechanism of myocardial stunning. DZX may therefore provide protection by maintaining myocyte volume homeostasis during stress. In this fashion, observations at the cellular level may provide mechanistic insight into responses at the organ level.

The exact mechanism of action of the K_ATP_ channel opener DZX has remained elusive. DZX may provide myocyte volume homeostasis during stress at the K_ATP_ channel itself or at a channel-independent location.^20–22^ K_ATP_ channels have been described on both sK_ATP_ and mK_ATP_ membranes. Opening of the sarcolemmal channel results in K^+^ efflux from the cell, and opening the mK_ATP_ channel is proposed to result in K^+^ influx from the cytosol into the mitochondria.

To attempt to localize the site of DZXs mechanism of action, a purported mitochondrial channel inhibitor, 5-HD, and a purported sarcolemmal channel inhibitor, HMR 1098, were separately added to hypothermic St. Thomas solution containing DZX. In addition, 5-HD was added to MI solution containing DZX. Neither channel inhibitor altered the observed decrease in cell swelling associated with DZX during exposure to stress. This is consistent with observations in animal myocytes.^1–4^ This finding may be related to the nonspecificity of channel inhibitors or it may support a K_ATP_ channel-independent mechanism of DZX.^23,24^ A non-sK_ATP_ channel location of action is also supported by definitive experiments using mouse whole-cell voltage clamp and sK_ATP_ channel subunit Kir6.2 knockout mice.^25^

The genetic structure of the sK_ATP_ channel has been determined; however, the structure of the proposed mitochondrial channel has not. Therefore, claims of channel opening and inhibition are often implied with use of pharmacological manipulation. The specificity of K_ATP_ channel openers and inhibitors has been challenged, introducing uncertainty to the isolated use of pharmacological manipulation.^23,24^ Alternative, K_ATP_ channel-independent targets of DZX have been proposed and will be the focus of future work.^26^ The determination of DZXs mechanism of action warrants further investigation and will be greatly aided by the determination of a mK_ATP_ channel genetic identity.

Significant myocyte swelling (2%) secondary to hypothermia alone (hypothermic control Tyrode's solution) was observed in this study. This swelling was much less than that observed following exposure to hypothermic St. Thomas solution (8%), hyposmotic stress (7%), or MI (6%). This observation is not consistent with previous observations in animal ventricular myocytes.^1–4^ The addition of DZX to hypothermic Tyr did decrease swelling due to hypothermia alone by 50%, which is consistent with the hypothesis that K_ATP_ channel openers play a role in myocyte volume homeostasis. However, this finding in human myocytes suggests that the additive effect of three stresses (hypothermia and ischemia and exposure to hyperkalemic cardioplegia as in cardiac surgery) could be detrimental to myocyte function. This clearly warrants further investigation.

In addition, human myocyte volume tended to demonstrate a prolonged increase in cell size during stress (hyperkalemic cardioplegia or osmotic stress) and a prolonged return to normal volume on reexposure to Tyrode's solution (particularly in the MI group) in contrast to animal myocytes and previous work in human myocytes^15^. Variations in isolation methods, species differences, individual patient comorbidities, potential differences in the speed with which the test solutions reached the myocyte bath, and other differences such as the use of human atrial myocytes versus the use of animal ventricular myocytes may account for these results.

### Study Limitations

Isolated myocytes are advantageous because they allow for repeated estimations of cell volume. This model is not intended to mimic the clinical situation of ischemia and reperfusion. This model allows for the independent evaluation of one stress at a time and a more careful evaluation of the effects of K_ATP_ channel openers. Caution must therefore be taken in applying these findings to the whole organ or organism level.

The solutions evaluated in this study were crystalloid solutions, which may result in myocyte swelling. However, any baseline myocyte swelling due to isolation procedures in this study would be evident in the control Tyr group. Comparisons to control crystalloid solution and the representation of all data as a percentage of baseline should correct for any direct effect of the solutions themselves. In addition, crystalloid solutions were used in order to be consistent with previous work using rabbit and mouse myocytes.^1–4^
